# Microbiome diversity is a modifiable virulence factor for cryptosporidiosis

**DOI:** 10.1080/21505594.2023.2273004

**Published:** 2023-11-10

**Authors:** Georgina. R. Hurle, Julii Brainard, Kevin. M. Tyler

**Affiliations:** Norwich Medical School, University of East Anglia, Norwich, UK

**Keywords:** *Cryptosporidium parvum*, microbiome, microbiota, dysbiosis, metagenome, meta-analysis, systematic review

## Abstract

*Cryptosporidium* spp. infection causes significant disease in immunosuppressed individuals and children under the age of 5 years. The severity of the pathological presentation of cryptosporidiosis is a function of the host and parasite genotypes, host immune status, and the enteric environment or microbiome of the host. Cryptosporidiosis often presents with abdominal pain and severe diarrhoea and is associated with intestinal dysbiosis and inflammation. Our systematic analysis of the available literature revealed that bacterial diversity is reduced during infection in larger animal models, lending support to recent studies which indicate that the use of probiotics or the presence of a naturally diverse gut microbiome can prevent or minimise pathology caused by gastrointestinal pathogens. In summary, we present evidence that the presence of a diverse gut microbiome, natural or induced, reduces both symptomatic pathology and oocyst output.

## Introduction

Cryptosporidiosis is a disease induced by the apicomplexan gastrointestinal (GI) parasite *Cryptosporidium* spp. The severity of cryptosporidiosis is a function of parasite genotype, host genotype, immune status, and enteric environment (microbiota) of the host [1,2]. This genus of parasites can be found globally, with two predominant species causing human disease: *Cryptosporidium parvum* and *Cryptosporidium hominis*. Recently, work by Nader *et al* (2019) found further distinction, describing sub-species, *C. parvum anthroponosum* and *C. parvum parvum* [3] which cause significant human disease. Cryptosporidiosis is particularly prevalent in developing countries, in populations with immunosuppressive conditions such as HIV/AIDS, and in children under 5 years of age [4,5]. *Cryptosporidium* infection can be either zoonotic or anthroponotic [3,6] and can enter the host via contaminated water or food sources [7]. Once in the gastrointestinal (GI) tract of the host, four motile sporozoites are released from each oocyst [8,9]. These sporozoites infect the intraplasmamembrane space of the epithelia lining the small intestine. The mechanism of cell invasion is not fully understood, one hypothesis suggests sporozoite contact results in the hijacking the host actin cytoskeleton, with another mode of entry thought to involve the relocation of a sodium/glucose cotransporter in the host membrane, which results in water influx to infection site and parasite entry to the cell before the relocalisation of the host membrane around the parasite [10]. Cell invasion facilitates nutrition uptake by the parasite and subsequent growth and proliferation in the intestine. Infection results in a cascade of symptoms, including watery diarrhoea, dehydration, muscle wasting, and in severe instances, can prove fatal [1]. Infections in immunocompetent individuals are self-limiting; however, children and immunosuppressed adults are at a high risk of developing severe disease.

As an infection of the GI tract, research has demonstrated the impact *Cryptosporidium* infection has on the composition of the gut microbiome. The composition of the gut microbiome and the ratio of certain species can have a significant impact on an individual’s quality of life [11–13]. Infection and use of antibiotics can result in dysbiosis of the gut. Dysbiosis can be defined as a compositional change in gut bacterial communities that results in a temporary lack of diversity, hallmarked by a rise in bacteria which can cause inflammation or bacteria with pathogenic potential [14]. The impact on the host has been shown to have potentially long-term effects, such as irritable bowel syndrome [11,15]. Additionally, a significant reduction of host bacterial diversity has been linked to a dulled immune response and the establishment of host gut microbiomes with potentially harmful bacteria [14,16]. The immune system has been shown to respond to commensal gastrointestinal bacteria via pattern recognition receptors (PRRs), toll-like receptors (TLRs), and nucleotide-binding oligomerisation (NOD)-like receptors [17,18]. These interactions can lead to intestinal inflammation [17–19]. As a result, the gut microbiota can drive an elevated or misdirected immune response against various pathogens, including *Cryptosporidium* spp., which affect disease severity [17].

Studies discussing gut diversity have used a variety of statistics to measure the composition of the host’s gastrointestinal microbiome [20]. The most commonly used diversity statistics are the alpha indexes Shannon and Simpson [21], equations shown in Table 1. These diversity index statistics measure species richness from the number of different species in a given dataset (Shannon index), and the evenness, or number of each species in a dataset (Simpson index). Alpha statistics measure the diversity of a specific area or ecosystem [24]. Statistics which look at the changes in diversity between ecosystems are denoted as beta statistics, which take into account the changes in abundance between ecosystem “A” and ecosystem “B” [25]. Taking this further, looking at the overall diversity of a large region or many ecosystems would require the use of gamma diversity indices [26]. As the gut microbiome is one community, alpha statistics are more commonly used than beta and gamma indices to measure diversity. However, a recent paper by Kers *et al* (2022) found the structure of a study can influence which alpha indices are most sensitive, and as a result provide a study with statistical significance which may not exist, should a more appropriate statistic be used [27]. This study also found beta-metrics such as the Bray-Curtis to be the most sensitive to observe differences between groups, as a consequence lowering bias in publication and is appropriate for use in studies with a lower sample size [27]. Nevertheless, this review is focussed on the Shannon alpha metric as a mode of diversity measurement because it is the more prevalent statistic used in microbiome analysis to date.

**Table 1.** Alpha diversity indexes and their corresponding equations.

	Diversity Index	Formula	Reference
Alpha Diversity Indexes	Shannon Diversity Index	$H=-\overset{s}{\underset{i=1}{\sum }}\left(p_{i}lnp_{i}\right)$	Lemos *et al* (2010) [22]
		*s* is the number of OTUs, *p*_*i*_ is the proportion of the community represented ay OTU *i.*	
	Shannon Reciprocal index	$\frac{1}{ln\left(H\right)}$	
	Simpson Diversity Index	$D=\frac{1}{\sum_{i=1}^{s}p_{i}^{2}}$	Simpson (1949) [23]
		*s* is the total number of species in the community, *p*_*i*_ is the proportion pf community represented by OTU *i*.	
	Simpson’s Reciprocal Index	$\frac{1}{D}$	

Experiments have focused on understanding the effects of microbiome composition on the outcome of enteric infections. These studies have endeavoured to establish whether the microbial composition of the GI tract is linked to resistance against infection or to the attenuation or exacerbation of infectious diseases, such as cryptosporidiosis. Most studies rely on the quantification and classification of bacterial taxa present in a variety of mammalian hosts prior, during, or after infection, and were conducted by sampling faecal matter or contents directly from the GI tract. To consider whether relationships could be observed between microbial diversity and composition from the existing publicly available metagenomic datasets, we aimed to determine whether alpha-diversity, a measure of species richness, recorded before, during, or after infection with *Cryptosporidium parvum* was associated with disease severity or trends in which bacterial phyla reduce, or increase in abundance, during, or as a result of infection [26]. In addition, we aimed to investigate risk factors that might affect the susceptibility and severity of the clinical disease course in relation to parasite output and diversity. We applied systematic review methods to find relevant literature which are described narratively. Systematic review methods are a comprehensive approach to evidence synthesis which can be designed to collect and evaluate evidence on a research question without imposing bias or expectations for final conclusions [28]; this is the lens with which we approached the literature related to the relationship between the gut microbiome and infection with *Cryptosporidium parvum*.

## Materials and methods

Data was analysed and gathered in two different ways to fulfil the aims of the review. These Aims were:

1. What impact does infection have on diversity of the gut microbiome during infection?
2. Does the composition of the gut microbiome affect cryptosporidiosis susceptibility or severity?

**Figure 1.**
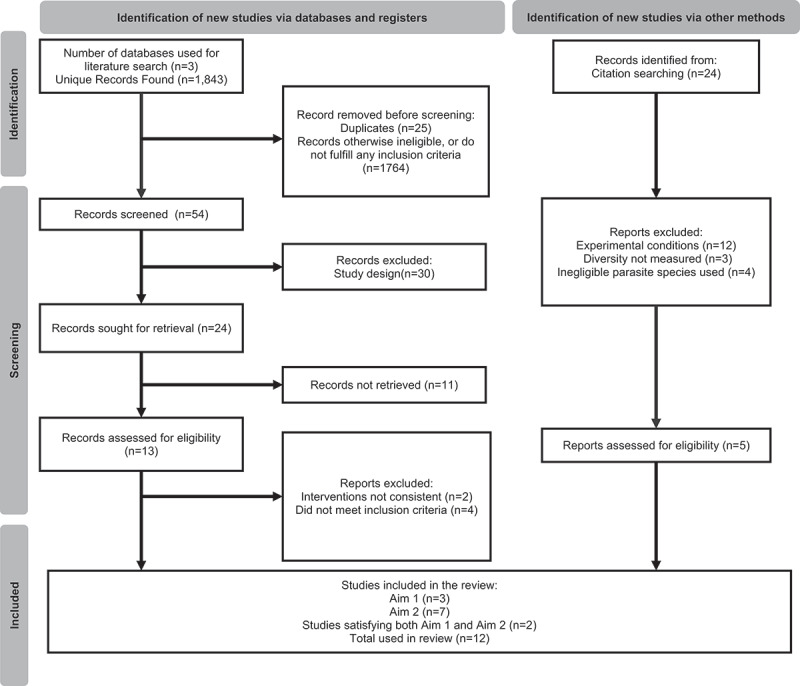
Overview of study selection process. PRISMA flow chart showing the article screening process from three databases: PubMed, Google Scholar, Web of Science.

To do this, we used independent inclusion and exclusion criteria to find literature best suited to synthesising the most accurate data for the review. Literature searches were conducted in October 2021, following the PRISMA search and reporting guidelines [29]. Three studies were included to inform analysis focussed on the relationship between cryptosporidiosis infection and bacterial diversity (Aim 1), seven studies were eligible to inform descriptive analysis in Aim 2 (Figure 1, Table 3). Two studies were found to satisfy both Aim 1 and Aim 2, and so were added to the analysis for each aim (Figure 1, Table 3). A total of twelve studies were used in this review, see Table 3 for details on which papers were used to inform each aim.

**Table 2.** Compiled Shannon index scores (*H*) from studies further described in Table 3.

Author (Year)	Subject	Symptoms	Treatment	Group/Animal Name	Infection Status	Shannon Index (*H*)
Ras *et al*. (2015) [30]	Adult CD-1 outbred mice	Not recorded	Immunosuppressed – Dexamethasone	Group 1 – MD *C. parvum* isolate	Uninfected	4.9
					Infected	5.1
				Group 2 – TU114 *C. parvum* isolate	Uninfected	5.0
					Infected	4.8
Charania *et al*. (2020) [34]	IL-12 KO C57BL16 mice	Not recorded	No treatment for groups used in this review		Uninfected	4.0
					Infected	3.7
McKenney *et al*. (2017) [33]	Coquerel sifaka’s – Captivity	Lethargy, anorexia, or diarrhoea	1dpi – Antimicrobials 7dpi – Antibiotics Received faecal Transplant	Primate-1	Uninfected	6.7
					Infected	5.0
			3dpi – Antimicrobials 8dpi – Antibiotics Received Faecal Transplant	Primate-2	Uninfected	8.0
					Infected	6.2
			1dpi – Antimicrobials 6dpi – Antibiotics Received Faecal Transplant	Primate-3	Uninfected	8.1
					Infected	5.9
			No Treatment	Primate-4	Uninfected	8.8
					Infected	7.5
			11dpi - Antibiotics	Primate-5	Uninfected	7.9
					Infected	6.0
			No treatment	Primate-6	Uninfected	8.0
					Infected	7.7
Mammeri *et al*. (2019) [31]	5 day old, Outbred, CD-1 mice	Not recorded	No treatment mentioned	Experiment 1	Uninfected	0.78
					Infected	1.0
				Experiment 2 – Repeat of Experiment 1	Uninfected	0.95
					Infected	1.46
Mammeri *et al*. (2020) [32]	21 day old, male, French Alpine goat kids	Watery, yellow faeces with clumps and mucus. Hypothermia, dehydration, growth deformities, weight loss and death.	No treatment	Control	Uninfected	9.5
				Infected	Infected	7.0

**Table 3.** Studies included in this review.

Aim Add ressed in Review	Authors	Date of Study	Accession Numbers	Participants (number of subjects)	Clinical Symptoms	Intervention (Length of Treatment)	Prior Antimicrobial Treatment?	Effect on Oocyst Shedding
1	Ras *et al* [30]	2015	PRJEB7853	Adult out-bred CD-1 mice (**30**)	-	No intervention	Dexamethasone 21-phosphate (**Day 8–2 days after first dose mice were challenged with *C. parvum***)	Study did not measure this outcome – focus was on bacterial diversity.
1	Mammeri *et al* [31]	2019	-	5-day old neonatal outbred CD-1 mice (**36**)	-	No intervention	No prior treatment	Study did not measure this outcome – focus was on bacterial diversity.
1	Mammeri *et al* [32]	2020	PRJNA603642	21 day old, male, French Alpine goat kids	Watery, yellow faeces with clumps and mucus. Hypothermia, dehydration, growth deformities, weight loss and death.	No intervention	No prior treatment	Study did not measure this outcome – focus was on bacterial diversity.
1 and 2	McKenney *et al* [33]	2017	SAMN06349027–SAMN06349170	Coquerel’s sifakas (**35**)	Lethargy, anorexia, or diarrhoea	Treatment varied between primates, listed are interventions used on the sifakas which received faecal transplants. Ampicillin (**6 days**), Ceftazidime (**6 days**), Metronidazole (**5.5 days**), nitazoxanide (**12.6 days**), ceftiofur (**5 days**) Faecal transplants originated from healthy donors.	None – Treatment given to specific animals during infection. See Table 2.	Faecal transplants resulted in resolution of symptoms. Diversity of faecal microbiome higher after transplant than pre-infection levels. Those without faecal transplants had reduced diversity after infection.
1 and 2	Charania *et al* [34]	2020	-	IL-12 KO C57BL/6 mice (**NumberUnknown**)	-	Cloxacillin (**1 day prior to infection**) Paromomycin (**1 day prior to infection**) Vancomycin-Imipenem (**1 week prior to infection**)	None	More oocysts shed in mice givencloxacillin, than vancomycin-imipenem Lower bacterial diversity in cloxacillin treated mice than vancomycin-imipenem and paromomycin treated mice.
2	Alak *et al* [35]	1997	-	C57BL/6 female mice (**40**)	Oocysts in faeces	*Lactobacillus reuteri* (**10 days prior to infection**)	LP-BM5 treatment	*L. reuteri* supplemented mice showed reduced oocyst shedding compared to controls
2	Pickerd and Tuthill [36]	2004	-	12-year old girl with Coeliac’s Disease (**1**)	4 months abdominal pain, flatulence, loose stools, nausea, lethargy.	*Lactobacillus* GG and *Lactobacillus casei* Shirota (**4 weeks**)	None	Diarrhoea and nausea resolved in 10 days
2	Oliveira and Widmer [37]	2018	PRJEB25162, PRJEB25164	CD-1 female mice (**16**)	-	Probiotics consisting of: *Lactobacillus* (9 species), *Bifidobacterium* (4 species), *Streptococcus thermophilus*, acacia gum, larch gum, galacto-oligosaccharide, L-glutamine, vitamin D_3_ (**1 day prior to infection**)	Dexamethasone 21-phosphate disodium (**7 days prior to infection**) Vancomycin and streptomycin (**6 days prior to infection**) Metronidazole (**6 days prior to infection**)	Mice given probiotics released 8 times more oocysts than control mice. No significant increase of probiotic bacteria in faeces.
2	Ichikawa-Seki *et al* [38]	2019	-	Neonatal Holstein calves, female (**20**)	Faecal score was measured. No additional symptoms listed.	No Intervention. Infections were naturally occurring.	None	Increase in *Fusobacterium* was associated with increase oocyst shedding, and an increase in faecal scoring.
2	Oliveira and Widmer [39]	2019	PRJEB31954, PRJEB31955, PRJEB31958, PRJEB31959, PRJEB31960	C57BL/6 female mice (**32**) CD-1 female mice (**22**)	-	No fibre diets (**5 days prior to infection**) Probiotics: *Lactobacillus* (9 species), *Bifidobacterium* (4 species), *Streptococcus thermophilus*, acacia gum, larch gum, galacto-oligosaccharide, L-glutamine, vitamin D_3_ (**1 day prior to infection**) Prebiotics (**5 days prior to infection**)	Dexamethasone 21-phosphate disodium **(5 days prior to infection**) Vancomycin and streptomycin (**6 days prior to infection**) Metronidazole (**6 days prior to infection**)	Mice given prebiotics or fibre diet shed fewer oocysts than those with no fibre diets. Increased oocyst shedding/severity of disease in mice with a reduced microbiome
2	VanDussen *et al* [40]	2020	E-MTAB-9100	HCT-8 Cells	-	Top 20 neonatal ICR mice metabolites (**24 hours**)	None	Capric acid, Monomyristin and Lauric acid reduced oocyst shedding. DHA, LnA and LA increased *C. parvum* prevalence
2	Karpe *et al* [41]	2021	-	3 week old, female, C57BL/6J mice (**20**)	-	No Intervention	None	Increase in short chain fatty acid synthesis, as well as D-amino acid retention in the gut. Increase in yeast proteins detected. Oxalate was accumulated in the liver of infected mice.

## Inclusion and exclusion criteria

The parasitic species of interest was *Cryptosporidium parvum*. Studies on other parasites or *Cryptosporidium* species were ineligible. Hosts used to examine parasite impact were considered relevant if studies were conducted in land mammals. Data reported in these studies were required to provide insight into the dynamics of the gut microflora during *C. parvum* infection.

## Exposure/intervention

While our study was not focused on specific interventions and the effect they can have on the gut microbiome during *C. parvum* infection, some interventions were relevant to the aims. Interventions had to be used in the analysis of the gut or faecal microbiome of infected individuals, and studies with interventions must also have investigated infections without interventions in conjunction with uninfected control populations. There were no limitations on when the intervention had to have begun, whether before or after *C. parvum* inoculation.

## Study design and comparators

Studies had to have conducted deliberate experiments that focussed on measuring bacterial abundance, Shannon or Simpson indices, or written population analysis. There were no limits on the date of publication or study location. Studies that were not in English or were not easily translated using Google Translate were excluded. Studies that were not fully accessible were excluded.

## Outcomes of experiments

To be eligible, studies had to address at least one of the following:

- Show the gut flora profile in hosts during infection and either pre- or post- *C. parvum* infection
- Evaluate how an intervention affects the gut microbiome during infection
- Assess severity of cryptosporidiosis in infected hosts
- Provide diversity statistics in their data analysis
- Provide open, publicly accessible metagenomic data

## Reference sources

The search focussed on peer-reviewed studies that were conducted in October 2021. The databases searched were: PubMed, Google Scholar, and Web of Science. Conference information was not searched. Grey sites were searched for additional background information, including the Center of Disease Control (CDC) and the UK Department for Food and Rural Affairs.

## Search strategy

Preliminary searches enabled a handful of studies to guide future searches using the aforementioned databases. The keywords from studies that met the desired outcomes for the experiments, were assessed and used in the formal search for further studies.

Search terms:

*Cryptosporidium*

AND

(Microbiome OR microbiota OR metagenome* OR 16S rRNA)

Literature found was forward searched to find other relevant studies; however, a limited number of additional studies were found. Web of Science was used for most of the forward searches.

## Study selection

All studies were screened and read by one investigator (GH), but aspects of the studies and data analyses were discussed with KT. Full texts were acquired where possible, with data selection performed after reviewing all relevant studies.

Efforts were made to include studies that provided full public access to metagenomic datasets. Articles that did not provide open access data under an accession number were emailed; four did not provide or respond to this information (Karpe *et al*. (2021), Ichikawa-Seki *et al* (2019), Mammeri *et al* (2019) and Charania *et al* (2020)). Accession numbers are listed in Table 3. Two additional studies did not provide metagenomic data due to the nature of the articles, and therefore did not have accession numbers; these are also shown in Table 3.

## Data synthesis

The composition of the gut microbiome and its effect on susceptibility was determined for each individual study. Then, the data was then combined. Pooling was only possible for comparable interventions (or lack thereof) and for identical organisms tested.

Diversity was analysed by compiling the diversity statistics used in the studies that fit the inclusion criteria. The Shannon diversity index (*H*) was used as a comparative statistic between the relevant studies. Shannon indices were extracted from, or were calculated from, studies that fit the relevant criteria for Aim 1 using Microsoft Excel. Differences in the Shannon Diversity Index for infected and uninfected animals were compared between species groups using the Mann Whitney U test, this statistical test was conducted using Stata (v. 18).

*H* was used to describe the likelihood of a particular species of bacteria occurring next in the dataset by combining species richness and the effective number of species in a sample [42]. A Shannon index of 0 indicates that there is no diversity in a given dataset, such as if the gut is dysbiotic, wherein the gut microbiome is dominated by one or two species. There is no fixed upper limit for *H*, as it is dictated by log(k). When all species in a dataset are evenly represented, *H* equals log(k), where k represents the number of species [42]. This would dictate the maximum Shannon index value, which, if reached, would be interpreted as a sample with infinite diversity. Therefore, the higher the *H* value, the more diverse the microbiome analysed across studies.

## Results

Table 2 shows the literature addressing diversity (Aim 1). All studies used in this review (both Aim 1 and Aim 2), their interventions, subjects used, and treatment are summarised in Table 3.

## Microbial diversity is lost during cryptosporidiosis in non-rodent models

**Figure 2.**
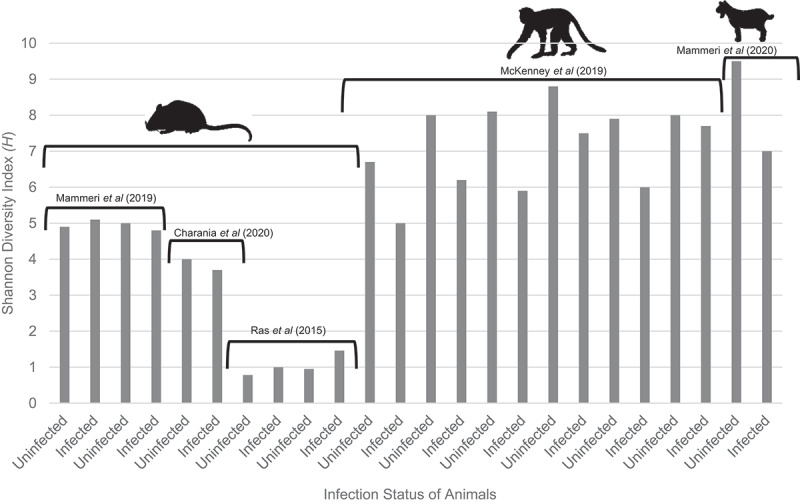
Shannon index scores (*H*) from studies described in Table 2. Shannon Index (H) values obtained from studies listed in Table 2. Order of the bars follows the order of Table 2 (descending) from left to right. Where specific Shannon Diversity Indexes could not be found within the study, the value was estimated from graphs provided within studies or the supplementary material. Mice studies indicated by mouse image, primate study highlighted with primate image, goat subjects indicated using a goat image. Images obtained from BioRender, then edited to remove colour.

The literature was reviewed to investigate specific changes in α-diversity of the faecal microbiome, using the Shannon index (*H*) as a comparator between animals, both before and post-infection with *C. parvum*. Typically, the higher the *H* value of a given sample, the greater the microbial diversity; the lower the Shannon index, the lower the diversity, indicating dysbiosis of the gut. From eligible studies, three animal systems were used: mice, goat kids, and primates. In most instances, active infection with *Cryptosporidium* resulted in a reduction in faecal microbiome diversity compared with pre-infection levels (Table 2 , Figure 2), with a decrease in bacterial diversity being highlighted, especially in primate and goat kid models, while experiments using murine models showed variable levels of *H* diversity.

The largest reduction in the *H* index was noted in the goat kid systems [32], with the next largest reduction occurring in Primate-3 in McKenney *et al*’s (2017) study [33]. Of the primates, only four were treated with antibiotics and antimicrobials, each showing different levels of diversity reduction after treatment. Treatment with antimicrobials or antibiotics in primates resulted in a maximum reduction of *H* = 2.2 (Primate-3), whereas primates receiving no treatment had a maximum reduction of *H* = 0.3 (Primate-6). This indicates that treatment of cryptosporidiosis with antimicrobials or antibiotics in the early infection stages results in a large loss of bacterial diversity compared with untreated primates.

A higher initial bacterial diversity does not necessarily equate to protection against *C. parvum* infections. This finding was highlighted in the primate population. Primates with high bacterial diversity prior to infection received more, or equivalent, treatments than primates with less diverse microbiomes prior to infection (Table 2). This being so, in this study there were two primates (Primates-4 and −6) which received no treatment. These specific primates had initial *H*-values of *H* = 8.0 (Primate-6) and *H* = 8.8 (Primate-4), which were similar to other primates in the study, such as Primate-3 (*H* = 8.1, prior to infection). This highlights the impact of *C. parvum* on the composition of the gut microbiome without antibiotics and antimicrobials, which could enhance the effect of infection on bacterial diversity. This indicates that *C. parvum* itself reduces bacterial diversity in the gut during infection, regardless of whether the infection is treated. Primate-3, however, was treated with antibiotics and antimicrobials, and received a faecal transplant. The specific composition of the faeces chosen for transplants to these three subjects was not analysed. The specific microbiomes in Primate-4 and Primate-6 may have contributed to the protective effects, which resulted in these primates not receiving treatment for cryptosporidiosis, unlike primate-3, which showed equivalent levels of bacterial diversity prior to infection, but received treatment. This indicates that the specific composition of the gut microbiome may contribute more to protection against infection than simply having a diverse array of bacteria in the gut.

Studies using mice as subjects to measure bacterial diversity have shown mixed results, with no differences observed in the Shannon index before or during infection. Mammeri *et al* (2019) recorded an increase (*H* = 0.2) in bacterial diversity between pre-infection and active infection status of mice infected with the MD isolate of *C. parvum*, whereas mice infected with the TU114 isolate of *C. parvum* showed a reduction (*H* = 0.2) in bacterial diversity. Mammeri *et al* (2019) used the same breed of mice as those in Ras *et al* (2015) study, outbred CD-1 mice. Ras *et al* (2015) showed a consistent decrease in the Shannon index between infected and uninfected experimental groups. Mammeri *et al* (2019) showed varied results, with both an increase and decrease in diversity recorded during infection. Charania *et al* (2020) reported results consistent with those of Ras *et al* (2015), in which a decrease in the Shannon diversity index was recorded after *C. parvum* infection.

Table 2 and Figure 2 summarise the changes in the Shannon Index of the gut microbiome in both infected and uninfected animal systems. Overall, mouse-based infection systems showed less diversity in terms of *H* than other animal models (Figure 2). When comparing the types of mice used in these studies, CD-1 mice showed variable alpha indices between the studies by Mammeri *et al* (2019) and Ras *et al* (2015), both of which used outbred adult mice. The only notable difference was the treatment, where Ras *et al* (2015) treated mice with dexamethasone (Table 2) prior to infection with *C. parvum*. This difference in mouse microbiomes between studies demonstrated the impact treatments can have on diversity, as well as natural variation between experimental groups from different institutions. The average decrease in Shannon diversity is greater for larger mammals than in mouse models. There was a significant (Mann Whitney U test, *p* = 0.006) difference between the average change in Shannon diversity between murine models (mouse, *n* = 4) and larger mammalian animals (primates and ruminant, *n* = 7) in infected and uninfected states. This significant difference in diversity could be attributed to numerous factors, such as diet and location. Primates and goat kids were on a less controlled diet than lab fed mice. In addition these goats and primates were also in a less controlled environment than lab-based mice and were subjected to different weather and temperature conditions. The primate and ruminant groups could have encountered other animals, or faecal samples could have been in contact with different physical environments prior to collection and DNA extraction. This less controlled environment and external animal contact may provide more opportunity for unique types of bacteria to colonise the gut, increasing GI tract diversity in the larger mammals than in laboratory based mouse models. Due to the small differences in diversity between mice it is difficult to draw meaningful conclusions regarding the impact of cryptosporidiosis in rodent models. Larger mammal models present more conclusive correlations between infection and Shannon diversity.

## Microbial diversity correlates with reduced susceptibility and improved outcomes for cryptosporidiosis

Table 3 shows detailed summary of interventions, subjects used and treatment for all studies included in this review. The first half of Table 3 summarises diversity literature used to answer Aim 1, the latter half of Table 3 summarising severity and susceptibility literature used to answer Aim 2.

Gut microbiome composition varies from individual to individual, with each member of the bacterial community releasing metabolites into the lumen of the host’s gastrointestinal tract. VanDussen *et al* (2020) found that long-to medium-chain fatty acids released from cells in the gut act as inhibitory factors for *C. parvum* growth in neonatal mice [40]. However, metabolites such as linolenic acid (LnA) and docosahexaenoic acid (DHA) released by members of the genera *Lactobacillus* and *Bifidobacteria* exacerbate infection in neonatal mice, resulting in worsening symptoms [40]. This trend was observed *in vitro*, where cultures supplemented with DHA or LnA were found to have more infections with *C. parvum*. In this case, the absence of a gut microbiome translates to increased susceptibility to *Cryptosporidium* spp. infection in the presence of DHA and LnA [34]. Karpe *et al* (2021) found that short-chain fatty acid production, specifically butanoate, was upregulated in mice infected with *C. parvum*, along with retention of amino acids in the small intestine [41]. D-amino acid retention corresponded to an increase in *Lactobacillus* and *Coriobacteriaceae* [41]. In *Cryptosporidium* spp. infected calves, Ichikawa-Seki *et al* (2019) found *Fusobacterium* to be the most abundant genus of bacteria in the faecal microbiome during infection [38]. The increase in *Fusobacterium* coincided with an increase in oocyst output as well as more severe diarrhoea. Mice retaining their native gut microbiome displayed less severe symptoms as a result of *C. parvum* infection (Charania *et al* (2020)), when compared with IL-12 KO C57BL/6 mice treated with antibiotics, including paromomycin and cloxacillin [34]. This suggests that the composition of bacteria inhabiting the gut can affect the severity of cryptosporidiosis progression.

Some of the included studies focused on prebiotics and probiotics and their impact on pathology associated with *Cryptosporidium* spp. infections [35,36,39]. Probiotic studies have shown varied results, with regards to infection prevention and reducing symptoms severity. All but one study administered probiotic or prebiotic products one week prior to infection [35,37,39]. The only study to administer probiotics after infection was in the case of Pickerd *et al* (2004), where long-term infection resulted in worsening cryptosporidiosis [36]. Oliveria *et al* (2018) administered a probiotic product containing bacteria from the genera *Bifidobacteria*, *Lactobacillus* and *Streptococcus thermophilus* [37]. Mice fed diets supplemented with this probiotic product showed the highest levels of *Cryptosporidium* associated diarrhoea compared with infected controls that did not receive the probiotic supplement [37]. In contrast, in an alternative study mice fed a diet supplemented with *L. reuteri* were found to have a significant reduction in oocyst shedding compared to controls [35]. In a case study by Pickerd *et al* (2004), a patient receiving *Lactobacillus* bacteria showed prolonged symptoms of cryptosporidiosis that resolved after diet supplementation with probiotics [36]. Prebiotic diet supplementation has been found to impact oocyst shedding in mice [39]. Mice fed a no-fibre diet were found to shed between 1.5–3.2 more oocysts than mice fed a medium-fibre diet [39]. Diet has been shown to have a potentially significant impact on the severity of cryptosporidiosis; however, the specific beneficial genera have yet to be identified. Additionally, although bacterial species used in probiotic treatment are listed (Table 3), these studies do not mention the concentrations of bacteria in the probiotic, nor do they mention if the product was purchased commercially or made “in-house.”

Overall, the presence and composition of the microbiome were found to affect the severity of symptoms, with diet having a significant impact on the ability of patients to defend against *C. parvum* infection in the gut. Absence of a gut microbiome or poor diversity induced by antibiotics seems to be associated with worse symptoms of the disease. However, it is vital to evaluate the role of each genus of bacteria in the recovery, prevention, and treatment of cryptosporidiosis. Alternative treatments, such as faecal transplants, can have a positive impact on parasite infection. Primates transplanted from healthy individuals during infection with *C. parvum* were found to recover much faster than those not receiving a transplant [33]. However, primates were given this treatment as the last attempt to clear infection. In this context, faecal transplants were effective in reducing the severity of disease and limiting symptom progression.

## Discussion

In this review, we investigated how cryptosporidiosis affects the diversity of the gut microbiome using the Shannon Diversity Index and how the composition of the gut microbiome can affect symptoms and severity of disease.

We found evidence of a reduction in gut microbiome alpha diversity (Shannon Index) during *C. parvum* infection in ruminants, primates, and humans. Interestingly, there has been a clear record of specific genera that increase during infection, but little is known about which bacterial genera are depleted during, or because of, infection. Key taxa that increased during infection included Firmicutes (recently reclassified to Bacillota) [41], Proteobacteria (recently reclassified as Pseudomonadota) [33], Protozoa (presence of *Cryptosporidium* spp.), and Actinomycetota [40,41].

Firmicutes are the main contributors to butyrate production in the gut microbiome environment. This phylum includes gram-positive *Lactobacillus*, *Clostridiales*, *Enterococcus* and *Lachnospiraceae*. Higher butyrate production is associated with robust, richer bacterial communities and reduced hydrogen sulphide production [43,44]. Firmicutes increases dramatically after birth [38,45], with a study tracking this specific phylum increase in 20 neonatal calves [38]. Members of the Firmicutes phylum are also hallmarked as key members of a “healthy” gut microbiome [46]. The ratio of Firmicutes to Bacteroidetes (recently reclassified as Bacteroidota), has been linked to maintaining homoeostasis of gut flora and can be used as an indicator of patient health [47]. An increased Firmicutes/Bacteroidetes ratio is associated with obesity in both humans and mice [47,48]. This ratio has not been linked with the severity of cryptosporidiosis but could be an interesting avenue to explore. Alternatively, Chappell *et al* (2016) indicated that faecal indole concentrations could be used as a biomarker for susceptibility to cryptosporidiosis [49]. In this study, patients with high faecal indole levels remained uninfected when challenged with *C. parvum*, whereas those with lower indole levels were susceptible to infection [49]. Bacteroidetes were found to reduce indole levels in faeces, suggesting the Firmicutes/Bacteroidetes ratio alongside indole concentration in faeces could be accurate biomarkers for cryptosporidiosis susceptibility.

While “healthy” bacteria phyla (with presumed low pathogenic potentials) such as Firmicutes were seen to decrease during crytosporidiosis, a host of pro-inflammatory bacteria were observed to colonise the dysbiotic gut. Bacteria such as *Proteobacteria* are known for their ability to colonise the gut during dysbiotic events, including diseases [50,51]. Enteric infection, such as the one caused by *C. parvum*, induce inflammation of the gastrointestinal system, which is further exacerbated by colonisation of the gut with pro-inflammatory bacteria. High proportions of *Proteobacteria* in the gut have been associated with metabolic disorders and inflammation and are a marker of dysbiosis in the gut [50]. An increased abundance of *Proteobacteria* during infection is an indicator that *C. parvum* infection decreases bacterial diversity in the gut.

In the included studies, changing the diet of mice and the use of probiotics decreased the severity of cryptosporidiosis [35,36,39]. Lactobacillus only probiotics decreased oocyst output, but a mix of lactobacillus, bifidobacter and streptococcus used in one study showed substantially increased output of oocysts [35–37] indicating that a combination of host type and composition of the probiotic are likely to dictate outcomes of probiotic interventions. In studies reviewing diet, mice fed high-fibre diets were found to shed fewer oocysts than those fed a no-fibre diet [39]. High-fibre diets or probiotics may replenish bacteria that are flushed from the gut during infection, preventing pro-inflammatory bacterial colonisation that would otherwise exacerbate the symptoms of cryptosporidiosis. Alternatively, such interventions may resupply the gut with bacteria that aid in coordinating the host immune response against infection [52]. High-fibre diets could encourage Firmicutes to populate the GI tract, as this phylum can convert fibre polysaccharides into metabolites, such as short-chain fatty acids, which are used by surrounding bacterial communities [53–55]. The fermentation of fibre in the gut is a marker for good gut health [55], the bacteria that inhabit this nutrient niche in the GI tract could prove useful as another indicator of gut health, similar to how the Firmicutes/Bacteriodetes is currently used as a marker of obesity. Overall, the published studies to date suggest that dietary changes or probiotics could be used to reduce cryptosporidiosis severity, but they do not underpin the clinical administration of these products, nor explore if these products could be used to treat infection rather than reduce severity of disease. Thus far, studies have pre-emptively supplied hosts with probiotics prior to infection suggesting these are to be used to reduce severity of infection with *Cryptosporidium* spp. More research needs to be done on the stage of probiotic administration, for example, pre-infection to reduce severity, post-infection to alleviate symptoms of infection, or as a method to prevent chronic gut disease following infection such as irritable bowel syndrome through reconstituting bacterial diversity.

The studies included were few in number, had a small number of subjects, and were limited in their metagenomic analysis. Several relevant studies have been published without the datasets being made available. Many studies have examined the composition of the microbiome during infection, but some have not gathered the starting composition or recovery of the microbiome after infection, as a result, the full dynamic behaviour and adaptation of the gut microbiome has not been fully explored. Statistical analysis was undertaken comparing Shannon diversity before and during infection, further analysis was not attempted due to the lack of available data for this review. Due to the wide array of hosts used between studies, deeper statistical analysis is difficult, especially between groups which are not directly comparable or large in number. More extensive studies in these host organisms would provide data which could better drive this research area. Additionally, no studies have examined the faecal microbiome of humans infected with *Cryptosporidium* wherein the focus was on bacterial fluctuations associated with pathology, bioinformatic analysis was attempted, or diversity statistics made available. Studies tended towards describing the populations of bacteria that significantly increased during infection, and away from analysing the populations of bacteria that significantly decreased, which makes it difficult to pinpoint which phyla were most diminished as a result of infection. Murine models were inconsistent regarding the effects of infection on dysbiosis, and this correlates with a relative lack of symptoms and pathology, suggesting careful consideration should be taken prior to adopting them as a model of human or livestock disease. The existing evidence base is not large and is limited in other respects. More studies in this area are required, especially those undertaking full compositional comparisons at multiple time points post-infection to compare with pre-infection control microbiomes in mammalian systems.

From the studies analysed in this review, we are able to infer that symptomatic infection of the GI tract with *C. parvum* results in a reduction of bacterial diversity; however, it remains unclear whether the composition or type of bacteria present are able to protect the host from the most severe symptoms of cryptosporidiosis. Additional studies are required to fully understand how microbiome composition can modulate the severity of infection, which specific genera may provide protection, and which may contribute to the exacerbation of pathology.

## Conclusion

Existing work implies that the compositional dynamics of the gut microbiome during cryptosporidiosis reflect the inoculum, time since infection, host genetics, prior exposure, immunity to the pathogen, host diet, and the effects from any pharmacological treatment applied. To date, no studies have evaluated whether microbiome composition is correlated with the severity of cryptosporidiosis. Most studies focus on the composition of the microbiome during infection but neglect to include the composition prior to infection or during recovery. Extending studies to include such data will provide useful insights into GI microbiome dynamics during infection, and better determine the impact of pre-infection bacterial composition on the severity of subsequent symptoms associated with cryptosporidiosis. Published results show conflicting data regarding Firmicutes and probiotic interventions, highlighting the need for further research in probiotic impact, when probiotics should be administered to be most effective, and their future role in cryptosporidiosis treatment or prevention. Currently, probiotics appear to have a positive effect when taken 1 week prior to exposure with *Cryptosporidium* spp. Exploring fibre fermentation, indole levels, the role of diet, as well as the Firmicutes/Bacteriodetes (Bacteroidota/Bacillota ratio) as a predictor of disease severity should also be further explored and validated. Additional research should focus on consequential hosts (humans and livestock) rather than on rodent models.

This review affirms that cryptosporidiosis leads to dysbiosis of the host gut microbiome, which is characterised by an increase in bacteria in the taxa Firmicutes (Bacillota), Proteobacteria (Psuedomoadota) and Actinomycetota. Increases in the populations of inflammatory bacteria from numerous taxa could induce further inflammation of the gut, impact recolonisation of the GI tract, or have long-term health impacts. In essence, diverse composition of the microbiome prior to infection does not necessarily protect against infection, but may reduce the severity of symptoms associated with disease; while probiotics are not uniformly effective at reducing oocyst output, they do show the most promise for potentially preventing severe infection in immunosuppressed individuals and subsequent infection of others should oocyst output be reduced.

## Data Availability

No primary data is included. All data reported and analysed is publically available and fully referenced in the Tables.
